# Promoting professionalism through humanities-based transformation

**DOI:** 10.1080/07853890.2024.2386039

**Published:** 2024-08-05

**Authors:** David J. Doukas

**Affiliations:** Program in Medical Ethics and Human Values, Tulane University School of Medicine, and IntegratedEthics Program Officer, Southeast LA Veterans Health Care System, New Orleans, LA, USA

**Keywords:** Medical professionalism, medical education, academic medical centers, hospitals, virtue

## Abstract

**Introduction:**

In the last two decades, academic medical centers in the United States have faced a new challenge, dealing with breaches of medical professionalism in their staff, house staff, and medical students. Medical education settings have largely directed their professionalism efforts toward responding reactively to negative outliers.

**Discussion:**

This paper contends that the warrant of medical education mandates a transformative path forward. While negative behavior must be responded to meaningfully, so, too, must positive role models of professional behavior be publicly lauded for their consequential culture change in their institutions, and promoted as positive role models. Further, the promotion of medical professionalism must be part of this culture by proactively engaging all learners and health care providers with medical ethics and humanities-based knowledge, critical thinking skills, and role modeling.

**Conclusion:**

Professionalism programs should be vested with the authority to implement an affirmative educational program intended to nurture and promote medical professionalism in each medical student, resident, fellow, and attending and utilize methods to that end employing both virtue and care ethics.

## KEY MESSAGES

Medical professionalism is the foundational concept grounded upon scientific- and humanities-based knowledge and skills, directed toward the promotion of patient benefit with the rejection of self-interest, delivered with excellence in comportment, and the adherence to a covenant of trust with society.

Medical educators who solely emphasize the detection and punishment of negative outliers are missing essential elements in promoting medical professionalism.

Medical professionalism should be comprehensively addressed through a systematic addressing of teaching fundamental knowledge, skills, and virtue, promote excellence in role modeling and mentorship, and the redress of those lacking insight in their professional conduct.

## Introduction

Medical professionalism has been promoted as the key fundamental concept in medical education for the past quarter century, yet without giving the educational community a common definition to operationalize it, nor sufficient assets to promote it adequately. The introduction of contemporaneous medical professionalism began with an educational mandate by the Learning Objectives for Medical Student Education-Guidelines for Medical Schools by the Association of American Medical Colleges (AAMC) in 1998 emphasizing an ethical requirement based upon both duties and virtues expected of the medical student [1]. This mandate was soon followed by the Accreditation Council for Graduate Medical Education (ACGME), the Liaison Committee on Medical Education (LCME), the Joint Commission, and the National Board of Medical Examiners (NBME), which all emphasized the essential requirement of medical professionalism rooted in ethical and humanistic tenets [2–5]. The fundamental problem was that there was no common language regarding the definition of medical professionalism. This need led to the formation of the Project to Rebalance and Integrate Medical Education (PRIME), with its many publications on medical professionalism education, including the seminal Romanell Report on medical ethics education [1,6–12] and the founding of the Academy of Professionalism in Health Care (APHC) in 2012 [13]. These important efforts culminated in common language that defined *medical professionalism as the foundational concept grounded upon scientific- and humanities-based knowledge and skills, directed toward the promotion of patient benefit with the rejection of self-interest, delivered with excellence in comportment, and the adherence to a covenant of trust with society* [6,14]. Having this common language is critical since it directs subsequent medical education program learning objectives, measurement metrics, and remediation methods. This definition is based upon the historical footing laid out by Abraham Flexner in his early twentieth Century writings on humanities-based collegiate education and on other contributions outside the Flexner Report [15,16].

The relevant concern is the nature of our collective efforts in assessing and promoting medical professionalism. There is a lack of evidence that supports which medical professionalism interventions are effective when the concentration of effort is on remediating negative outliers [17]. A national survey on medical professionalism programs notes that evaluations noting the binary presence vs. absence of professionalism is both wanting and stultifying to the environment [18]. The behaviors cited in the survey are arrayed in four domains of lapses of involvement, introspection, communication, and integrity. Pervasive barriers to evaluation by faculty were noted such as lack of observation, time, training, how the system works, and plans to follow up [18,19]. An additional national survey noted challenges due to unclear policies, ineffective mediation, lack of training by faculty, and a reluctance by students and faculty to make reports, despite their strengths of promoting early intervention, promoting of professionalism generally, helping/not punishing students, and facilitative communication [20].

As discussed below, if we only stress remediation, our punitive basis for medical professionalism education fails to nurture the very thing we seek to amplify. Yet, the promotion of professionalism in AMCs has been advocated by some through a formal curriculum, ingrained throughout the curriculum and in each course, with clear educational metrics [21,22]. Nevertheless, a national curricular or system-based response has not been implemented for student, house officer, or faculty professionalism promotion of responding to deficiencies exhibited.

Efforts solely directed toward ‘fixing’ negative professionalism outliers miss the mark of education broadly. A programmatic redirection of efforts is required in all medical education venues that articulate the ethical and humanities-based aspects of knowledge and skills that are required for our learners, while also promoting excellence in role modeling through the cultivation of those virtues that were identified a quarter of a century ago by all of our medical education entities.

## Discussion

To date, medical professionalism has been evaluated in Academic Medical Centers (AMCs) at the point of the learner. The LCME evaluates educational programming in medical schools and toward medical students, the ACGME evaluates resident and fellow education and skill development, and the Accreditation Council for Continuing Medical Education (ACCME) evaluates continuing medical education to mitigate conflict of interest [23–25]. In all three domains, medical professionalism is the keystone of appraisal of the general competencies through sound medical ethics and humanities education and excellence in role modeling. However, the requirements for educational content, quality, and quantity of professionalism education are set by the school/program. As a result, the program can set its target outcomes (however low or high *they* choose) and then convey to accreditors that those outcomes have been met.

For schools and programs with truly high aspirations in professionalism formation education their metrics may be noteworthy, but regrettably programs may instead apply *de minimis* standards (for example, in the author’s experience interacting with clerkship and residency directors responding to simple ‘yes/no’ checkboxes to tick off on a variety of learner professionalism behaviors with no reflective aspect of evaluation are not uncommon). Similarly, one finding of a major survey concludes that a simplistic binary query gravely sells short the expansive reflection that professionalism evaluation is entitled [19]. Given the critical nature of medical professionalism towards professional outcome at all phases of medical education, it would be prudent for there to be aspirational national standards for ethics, humanities, and professionalism education in medical school, residency, and CME venues. Additionally, as there is a non-reciprocal nature of professional evaluation, AMCs should evaluate outcomes not only for their learners, but also for their teachers, directors, and administrators. Faculty of medical schools and residencies as well as directors, chairs, and Deans should be evaluated for their ethical and professional conduct toward their peers and learners. These educational leaders are responsible for setting the cultural tone of professionalism that exists at each AMC.

One mechanism for AMC review of medical professionalism currently exists: The Clinical Learning Environment Review (CLER) Program through ACGME [26], addresses six key areas of Patient Safety; Health Care Quality; Teaming; Supervision; Well-Being; and Professionalism. The ACGME-CLER visits may be helpful at considering the cultural state of AMC patient safety, quality improvement, and reduction in health care disparities. While CLER visits may provide an overview of the clinical operations and several key areas of learning environments, they may be insufficient in evaluating deficiencies in leadership and cultural integrity through the assessment of residency directors, faculty, and academic deans, and rectifying their shortcomings. CLER’s focus therefore misses the mark of safeguarding ethics and professionalism in faculty and leadership.

The recent 2022 CLER report [27] states that clinical learning environments ‘appeared to focus on addressing individual behaviors rather than measuring or assessing the overall culture of professionalism’. Additionally, these environments ‘uncommon[ly]…use and/or develop instruments to assess the culture of professionalism [27]’. Some may assume that directors of AMCs having ethics programs possess authority to address their professionalism culture – but that is fallacious. While ethics programs, departments, centers, and institutes commonly may have *responsibility* for teaching at their AMC’s medical schools and residencies, they very likely have no *authority* to respond, address, or act on faculty, administrative leadership, or AMC-culture aberrancies of professionalism. Ethics programs and their directors who have responsibility for ethics existing in the curriculum are very likely powerless to address deficiencies caused by the hidden curriculum and when pervasive toxic behaviors by individuals undermine academic medical centers and hospitals.

Medical education currently stresses ethics toward professionalism (and to a lesser degree humanities and role modeling) needed to become a medical provider, but students and practitioners can fall short in their behavior to peers and patients. Some anonymized real examples:

First, some short takes: “A **ranted** about his superior… B **cursed** at a resident… C is **cruel** and **abusive** to fellow students… D is **condescending** to her residents… E is **bullying** and **hostile** to nursing staff… F made **disparaging** remarks about her patient.”

Now, three brief anonymized case examples: “I am writing about a fellow medical student who has reported late to their clerkship morning shift every day this week. Our resident told all of us when to arrive for our shifts, yet this student would show up an hour or more each day causing stress on the other students – that is not fair to the other students who do show up on time.”

I am writing about a faculty member, Dr. A, whose comments in describing female and underprivileged patients were inappropriate and insulting. My student colleagues and I were aghast by this physician’s insensitivity.

I am very concerned as a resident about the attending Dr. B. who has displayed inadequate medical knowledge and acumen in treating specific types of patients. I observed one patient and this physician provided treatment that I believe was inadequate, while providing insufficient oversight for their fellow in this complicated case.

Such unprofessional behavior has spurred AMCs to designate and implement so-called professionalism programs. These programs tend to fall into two categories. First, there are top-down or peer review processes that are housed in Dean’s Offices in medical schools or Chief of Staff offices in hospitals. These can be reactive and depend upon their own experience and wisdom in formulating a unique response. Rarely, some AMC professionalism programs may even have authority to address *reported* deficiencies in the hidden curriculum or address *known* faculty integrity issues.

Second, there are critical-response entities addressing primarily negative conduct whose primary function is informational, and with a secondary function of utilizing administrative interventions within the organization’s hierarchy. The AMC will conduct an information exchange as delivered by a peer messenger in a ‘cup of coffee’ as a dispassionate conveyance of the information conveyed to the AMC by the complainant [28]. Reflection and self-correction by the recipient are the intended response. If the offending individual repeats more than three times with repeated unprofessional behaviors through the complaint collection system, then the circumstance would be met with escalating responses by their division chief, department chair, and dean level review with discussion and possible remediation measures.

However, medical professionalism (and their programs) cannot be sustained solely by focusing on the conduct of negative outliers. If an individual (i.e. teacher, resident, or medical student) falls short resulting in an outcome of unprofessional conduct, who precisely has failed? The person who exhibits the behavior, or the system that has recruited, hired, and perhaps even shielded this individual? Educational backgrounds and levels of character/maturity for learners and practitioners vary. We cannot be surprised by professionalism outliers if they previously have received a dearth of this requisite education, skill building, and role modeling. When we focus on negative role-exemplars, we may demean them to scapegoat status. While not a public humiliation, the process of a professionalism intervention may likely impart shame on the individual and may unfavorably reflect the culture of the AMC. Instead of solely depending on this negative feedback system, there needs to be an affirmative emphasis on teaching and cultivating the necessary knowledge and skills of professionalism, while also promoting positive role models who exhibit laudatory professional conduct and can thereby serve as mentors in the AMC. But how can this be done, through what infrastructure, and using what resources?

Consequently, AMCs require the tools and authority to conduct comprehensive evaluation of faculty, leadership, and culture to promote AMC professionalism. However, the current ability of AMC professionalism programs to respond may be hobbled by lacking the necessary authority, skills, and faculty in a response using an established moral framework. It is proposed that medical professionalism evaluation programs are best positioned, and should be vested with the warrant, to address these issues. Some AMCs already have excellent professionalism education with sound curricula in medical ethics, humanities, and role modeling, with identifiable Institutes, Centers, and Programs, or even Associate Deans for Professionalism, with faculty trained in health care ethics, humanities, and professionalism. Regrettably, these programs are the exception in American health care pedagogy, not the rule. Despite requirements for professionalism and ethics in medical schools by LCME, in residencies by ACGME, and in continuing medical education by ACCME, the metrics are on outcome metrics for each population *as set by the AMC* (noted previously). What these accreditation entities have not yet provided is the authority to promote medical professionalism with concrete, required aspirational guardrails articulating national standards of knowledge domains, skills, and hours taught, and expectations regarding the attendant resources in teaching and assessing professionalism outcomes.

We have reached a point in US health care education that we need to move beyond the simplistic punishment-only response to medical professionalism. What is now necessary is a systematic establishment of AMC professionalism programs transcending identifying negative outliers on the professionalism spectrum. AMCs now need the warrant and requisite personnel to take on the affirmative work of promoting positive role models by requiring 360-degree evaluations for excellence in displayed ethics and professional comportment and requiring administrative purview of coordinating all education toward professionalism by AMC professionalism programs to all learners and health care workers. The effort of public plaudits for persons displaying virtuous conduct can then be held out as an opportunity to learn from role models in one’s own institution, and to teach learners and providers who surround these individuals. Excellence in professionalism education should be the aspirational primary goal in health care education.

The current state of medical professionalism requires that professionalism formation education must raise its standards. Ideally, there should be an accreditation multi-organization-sponsored effort (i.e. ACGME, LCME, and ACCME) that moves beyond milestones and other outcomes-based criteria to identify a core curriculum toward professionalism, with corresponding measurable endpoints, that would be implemented longitudinally throughout medical school, residency, and continuing medical education. This curriculum would not only teach core concepts of knowledge in ethics and humanities towards professional care of the patient but would also have a required phase of learning in each setting to have a learner-mentor dyad to promote role modeling and mentorship. The transformation of AMCs is predicated on each institution having a designated professionalism program and director that will have ‘Inspector General’-type authority and purview regarding the professional conduct and education of all its students, faculty, and administrators.

To address medical professionalism education programmatically, a critical response has been advocated to implement curricula based upon virtue and care ethics towards professionalism [29]. Many of the complaints forwarded to professionalism programs are due to an evident exhibited lack of instilled virtue. It is prudent, then, to examine how virtuous character is an implicit requirement for professionalism. The prospect of virtuous behavior is hinged upon persons having virtuous character (as opposed to acting due to fear of consequences of a rule or law). The virtuous person *wishes* to act virtuously and will do so for that exact reason – to be virtuous [30]. Virtue (Greek: αρετή) means ‘excellence’ and is a form of knowledge identified with wisdom, portrayed through conduct, and enhanced through practice and repetition [31]. The virtuous person finds it easier to act virtuously, as a person literally changes their emotional characteristics to conform with excellence to the task at hand, like a trained athlete [32].

How do we change the virtues imbued in our medical learners? We must first acknowledge that we are all imperfect, and that we all can improve. We can each aspire to have the highest character that will result in excellent medical professionalism. We can all seek peers and mentors that can promote our moral growth. One may ask: ‘Can we not just select ‘paradigms of virtue’ as our learners by seeking out and selecting the most virtuous for entry into medical school, residency, or hospitals?’ First, as we are all imperfect, none of us is the perfectly chiseled sculpture of virtue. But we can seek out those learners and colleagues who have been noted by peers and teachers for aspects of character that will lend themselves to the healing arts. The Association of American Medical Colleges (AAMC) [33] has encouraged a set of premed competencies (including ‘ethical responsibility to self and others’, ‘empathy and compassion’, as well as nine other aspects of social, communication-based, and other pre-professional competencies to evaluate student readiness for medical school entry). Second, inasmuch as we cannot force feed virtue into people, we can teach and role model peers and learners professionalism, applying not only deontology, but also virtue and care ethics. But medical educators have had a hard enough time teaching the former, with little attention to the latter.

If we were to look at current curricula in medical education, the question is: Are Pellegrino’s [34] cited clinical virtues readily apparent in both content and outcomes: Compassion, Fidelity to Trust, Fortitude, Integrity, Justice, Phronesis/Practical Wisdom, Self-Effacement, and Temperance? Regrettably, despite some ambitious outliers cited by Doukas et al. [35], the predominant lack of virtue and care ethics education requires the next step to be taken by the AMCs. Professionalism is integral to the development of our learners and ourselves, with virtue and care ethics a means to achieve this goal. AMCs must nurture professionalism with both positive and negative reinforcement, designing curricula that will best encourage the best in us, rather than allowing us to portray the worst of us. However, it is still far better for AMCs to track and redress shortcomings rather than waiting for calamitous state board of licensure hearings or malpractice court cases.

The extant published literature is not expansive, yet there are representative professionalism curricula that emphasize medical schools, others that highlight residency and fellowship education, and other offerings that promote faculty development in professionalism and Interprofessionalism [36–42]. While these serve as helpful starting points, the integration of virtue and care ethics is the next step. In a recent scoping review evaluating the contribution of virtue and care ethics to professionalism and humanism education, a thematic construct was offered toward enhancing professionalism evaluation identifying how normative factors, such as virtue development, altruism, and care, are coupled with clinical factors of pedagogy and role modeling, resulting in humanistic behavior/praxis [29]. As the learner advances, there will likely be significant impediments such as the dissonance that occurs between virtue and Principlism teaching, as well as the challenges of dealing with negative influences of the hidden curriculum [29]. The oft-asked deontological question, ‘What is right or obligatory to do?’ is not the *only* query in health care. We must consider the character of the moral agent and consider how to address gaps. ‘How should we *be*?’ matters because it is only by becoming better people that we will do the right thing, and providers and learners of high character will better operationalize deontological ethics and critical thinking skills in medical humanities to serve the patient. What follows is a thematic structure suggested by these writings gleaned from this scoping review to allow each educational venue to customize their professionalism curriculum.

AMCs medical professionalism programs need guidance and structure on how to enhance professional conduct with a pathway for both short- and mid-term goals of implementation. A framework drawn from the scoping review’s resultant citations is proposed to address three levels of professionalism formation: *Roots, Knowledge, and Skills* [29]. In the *Roots* component, the initial requisite short-term goal in this process is addressed by the identification of aspirational goals and gaps in medical ethics and humanities education in learners and practitioners. For the educational system of the AMC, but also for those individuals for whom professionalism lapses have occurred, the identification of teaching gaps is intended to encourage promotion of ethics and humanities content for both the system and for the individual outlier. Additionally, the challenging task of role modeling in all medical education should be promoted with the intention of nurturing mentorship and relationships while addressing necessary components of competency-based professionalism. The Roots component would also address how education in professionalism communities nurture socialization as an intrinsic aspect of how the individual grows within the social contract that is being entered.

The Roots component is then followed by two mid-term goals in professionalism evolution. The second component of *Knowledge* identifies the specific key concepts of virtue and care ethics required toward professionalism formation: The fundamental concepts included are altruism in medical care, how the ethics of care is an intrinsic aspect of virtuous healthcare, how one can promote virtuous traits within oneself by knowledge, role modeling, and self-learning in this area. AMC professionalism programs should coordinate this rigorous educational pedagogy to all students, residents, and staff physicians, providing excellence in quality and quantity of virtue ethics education and role modeling, with the promotion of Academy-based teaching for its teachers as well as promotion of excellence and role modeling. AMCs are responsible to address issues of the influence of the hidden curriculum. They must mitigate negative aspects of social past anti-virtue conduct embedded within its system and nurture character formation as part of one’s professional duty beyond deontological duty.

The third component for AMCs to nurture is *Skill Building*, which concerns relationship cultivation throughout the educational spectrum. The outcome of professionalism is the product of the connections each has to others through humanistic behavior. Besides there being a fundamental social need for humans to peaceably interact, this factor is where being the recipient of excellent role modeling conveys to each student the required Hippocratic obligation of passing on medical education to the next generation, and the need to promote interprofessionalism. This also harkens back to the essential ‘root’ of professionalism communities to nurture peer relationships. Having both peer relationships and mentorship relationships flourish facilitates a fuller social contract model of medical professionalism.

Addressing potential challenges must always be considered with any new curricular proposal. Faculty to teach and serve as role models may not be sufficient in number or in training to teach the ethics and humanities content toward medical professionalism. To develop a new cadre to help implement these changes, faculty-teachers may be recruited from core clinical clerkship and residency domains of Family Medicine, Internal Medicine, Neurology, Obstetrics, Pediatrics, Psychiatry, and Surgery into a teaching academy faculty development program. Faculty would train using readily available in-person or online (live) graduate Certificate programs in Bioethics and Medical Humanities. This cadre of faculty learners would concentrate on the domains of clinical ethics, research ethics, and medical humanities that promote professionalism.

Role modeling is the other essential teaching academy area of concentration. This educational goal is not regularly promoted at all AMCs, and indeed role modeling has been deteriorating rather than improving recently [43]. Additionally, faculty may find it difficult to serve as role models if they themselves did not witness aspirational mentors in their own educational development. It is recommended that institutional support promote teaching academies that foster professionalism knowledge and skill growth of faculty, coupled with mentor-mentee pairs as well as peer support teams to encourage role modeling growth.

One question that arises pertains to future advances: Can medical professionalism bear up under the stresses of new scientific change? [44] The teaching of professionalism is accomplished by building critical thinking skills through medical ethics and humanities. This core knowledge allows for the learner to utilize frameworks such as deontology and virtue ethics using these critical thinking skills to address ethical dilemmas before them, while also affording them tools to explore the nuances of new scientific change. A similar argument was asserted at the initiation of the Human Genome Project regarding the potential of new ethical quandaries raised: Provide the analytical and humanistic skills, and they will allow the physician to make a cogent response even when technology leaps forward [45]. Teaching the fundamentals of professionalism will allow for the learner to adapt to technological and scientific breakthroughs that today’s medical student will see over the next 40–50 years of their lives. One of the greatest challenges that will ultimately make or break the future of professionalism is having AMCs support the economic cost of training learners and faculty in the necessary knowledge and skills toward becoming a physician. AMCs should move beyond the economic bottom line to see the great savings that professionalism offers both in terms of reducing unprofessional conduct by its practitioners (with its resultant adverse state board licensure and malpractice consequences) but also by promoting each physician to be an agent of change to advocate for our vulnerable and disadvantaged populations [46].

## Conclusions

AMCs are at a critical point of evolution in realizing medical professionalism. Not all AMCs may be starting at the same place, but each is attempting to do the same work. To nurture professionalism for all our learners and faculty, we need to do more than address negative outliers of professional conduct. We need to also commend publicly those who are positive role models for all learners and teachers, and each AMC should have a program that is given the warrant to oversee a comprehensive educational medical professionalism program that will ensure professional formation to secure the fulfillment of the social contract.
